# Genetic Polymorphism of Vitamin D Family Genes *CYP2R1*, *CYP24A1*, and *CYP27B1* Are Associated With a High Risk of Non-alcoholic Fatty Liver Disease: A Case-Control Study

**DOI:** 10.3389/fgene.2021.717533

**Published:** 2021-08-16

**Authors:** Minxian Wang, Ru Zhang, Min Wang, Liuxin Zhang, Yajie Ding, Zongzhe Tang, Hongliang Wang, Wei Zhang, Yue Chen, Jie Wang

**Affiliations:** ^1^Department of Fundamental and Community Nursing, School of Nursing, Nanjing Medical University, Nanjing, China; ^2^The Affiliated Brain Hospital of Nanjing Medical University, Nanjing, China; ^3^Ninghai Road Community Health Service Center of Nanjing, Nanjing, China; ^4^Department of Epidemiology, Shanghai Cancer Institute, Shanghai, China

**Keywords:** non-alcoholic fatty liver disease, cytochrome P450 family 24 subfamily A member 1 gene, cytochrome P450 family 27 subfamily B member 1 gene, polymorphism, risk

## Abstract

**Background:**

Previous studies have highlighted the important role of vitamin D and calcium pathway genes in immune modulation, cell differentiation and proliferation, and inflammation regulation, all closely implicated in the pathogenesis of non-alcoholic fatty liver disease (NAFLD).

**Objective:**

This study aims to investigate whether 11 candidate single nucleotide polymorphisms (SNPs) in vitamin D and calcium pathway genes (*CYP2R1*, *CYP24A1*, and *CYP27B1*) are associated with the risk of NAFLD.

**Methods:**

In this case-control study, a total of 3,023 subjects were enrolled, including 1,114 NAFLD cases and 1,909 controls. Eleven genetic variants in *CYP2R1*, *CYP24A1*, and *CYP27B1* genes were genotyped. Logistic regression analysis was used to assess the effects of these variants on NAFLD risk. The functional annotations of positive SNPs were further evaluated by bioinformatics analysis.

**Results:**

After adjusting for age, gender, and metabolic measures, we identified that *CYP24A1* rs2296241 variant genotypes (recessive model: OR, 1.316; 95% CI, 1.048–1.653; *p* = 0.018), rs2248359 variant genotypes (recessive model: OR, 1.315; 95% CI, 1.033–1.674; *p* = 0.026), and *CYP27B1* rs4646536 variant genotypes (additive model: OR, 1.147; 95% CI, 1.005–1.310; *p* = 0.042) were associated with an elevated risk of NAFLD. In combined effects analysis, we found that NAFLD risk significantly increased among patients carrying more rs2296241-A, rs2248359-T, and rs4646536-T alleles (*p*_trend_ = 0.049). Multivariate stepwise analysis indicated that age, visceral obesity, ALT, γ-GT, hypertriglyceridemia, hypertension, low HDL-C, hyperglycemia, and unfavorable alleles were independent predictors of NAFLD (all *p* < 0.05). The area under the receiver operating characteristic curve was 0.789 for all the above factors.

**Conclusion:**

The polymorphisms of vitamin family genes *CYP24A1* (rs2296241, *CYP24A1*, and rs2248359) and *CYP27B1* (rs4646536) were associated with NAFLD risk in Chinese Han population, which might provide new insight into NAFLD pathogenesis and tools for screening high-risk population.

## Introduction

Non-alcoholic fatty liver disease (NAFLD) has emerged as a major chronic liver disease, afflicting more than one-quarter of adults worldwide (Younossi et al., 2016). Previous studies have shown that NAFLD cannot only cause liver-related complications, such as non-alcoholic steatohepatitis (NASH), cirrhosis, hepatocellular carcinoma (HCC) (Lindenmeyer and McCullough, 2018) but also increase the risk of other extrahepatic diseases, such as cardiovascular disease (CVD) (Targher et al., 2010), type 2 diabetes mellitus (T2DM) (Tilg et al., 2017), and chronic kidney disease (Byrne and Targher, 2020). Its high global prevalence and poor prognosis have made NAFLD a serious public health threat.

The mechanisms underlying NAFLD are far from clear (Tarantino et al., 2019). Previous studies have indicated that NAFLD is a multi-factorial disease associated with a high frequency of metabolic comorbidities (Italian Association for the Study of the Liver (Aisf), 2017). Experts have reached a consensus on suggesting to rename NAFLD as metabolic-associated fatty liver disease (MAFLD), a more appropriate overarching term (Eslam et al., 2020). Dynamic interactions among insulin resistance, lipid metabolism, genetic variation, and other environmental factors shape the susceptibility and progression of this disease (Buzzetti et al., 2016; Eslam and George, 2016).

Vitamin D mediates immune-inflammatory and metabolic processes (Charoenngam and Holick, 2020; Szymczak-Pajor et al., 2020). Numerous studies have found that vitamin D deficiency is closely associated to the occurrence or development of insulin resistance-related diseases, such as T2DM, metabolic syndrome, and NAFLD (Cimini et al., 2017; Dawson-Hughes et al., 2020), despite its well-established roles in calcium homeostasis and bone mineralization (Reid et al., 2014; Borel et al., 2015). The local activated form of vitamin D is 1,25-dihydroxy vitamin D3 [1,25(OH)2D3]. Many diseases arise from low serum level of 25-hydroxyvitamin D3 [25(OH)D3], rather than vitamin D (Gandini et al., 2011).

The activation of 1,25(OH)_2_D_3_ requires two sets of hydroxylation. Vitamin D hydroxylases play a prominent role in this process (Höbaus et al., 2013). Vitamin D3 (cholecalciferol) is formed by cholesterol in the skin after exposure to sunlight. On the one hand, vitamin D3 is first hydrolyzed and synthesized into 25(OH)D_3_ and further catalyzed by 25-hydroxylase encoded by cytochrome P450 family 2 subfamily R member 1 (*CYP2R1*) and cytochrome P450 family 27 subfamily A member 1 (*CYP27A1*) genes. 25(OH)D_3_ is then hydroxylated in the kidney and synthesized into 1,25(OH)_2_D_3_ in other organs, which is catalyzed by the 1α-hydroxylase encoded by cytochrome P450 family 27 subfamily B member 1 (*CYP27B1*) gene. On the other hand, 1,25(OH)_2_D_3_ can be decomposed by 1,25-hydroxyvitamin-D3-24-hydroxylase encoded by cytochrome P450 family 24 subfamily A member 1 (*CYP24A1*) gene, maintaining the balance of vitamin D metabolism *in vivo*. Several genetic studies have reported that common single nucleotide polymorphisms (SNPs) in vitamin D pathway genes are associated with low-level serum 25(OH)D, including vitamin D receptor (*VDR*), vitamin D-binding protein (*VDBP*), *CYP2R1*, *CYP24A1*, and *CYP27B1* (Bedogni et al., 2006; Angulo et al., 2007; Wang et al., 2010; Boyle et al., 2012; Visscher et al., 2012; Zhu and DeLuca, 2012; Dastani et al., 2013; Braithwaite et al., 2015; Hibler et al., 2015; Woods et al., 2015; Jolliffe et al., 2016; Jung and Shin, 2016; Cheng et al., 2017; Thacher and Levine, 2017; Gibson et al., 2018; National Workshop on Fatty Liver and Alcoholic Liver Disease, Chinese Society of Hepatology, Association et al., 2018; Arai et al., 2019; Cho et al., 2019; Younossi, 2019; Zhang et al., 2019; Liu et al., 2020; Ma et al., 2020; Qian et al., 2020; Torkko et al., 2020; Yu et al., 2020). Genome-wide association studies (GWAS) have also identified that variants near these genes are associated with vitamin D status (Wang et al., 2010; Visscher et al., 2012). Moreover, previous studies have suggested a role of VDR, VDBP gene polymorphisms in the progression of liver diseases such as inflammation, liver fibrosis (Jung and Shin, 2016; Arai et al., 2019). Therefore, we hypothesized that the polymorphisms of these hydroxylase gene might regulate serum VD level and related biological processes. However, limited evidence can prove the influence of vitamin D family genes (*CYP2R1*, *CYP24A1*, *CYP27B1*) polymorphisms on NAFLD susceptibility.

The aim of this study was to investigate the correlation of candidate SNPs in vitamin D and calcium pathway genes (*CYP2R1*, *CYP24A1*, and *CYP27B1*) with the risk of NAFLD among Chinese Han population. Our findings may deepen our understanding of NAFLD and provide strategies for the screening, prevention, and individualized treatment of NAFLD patients.

## Materials and Methods

### Study Participants and Design

The participants of this case-control study were recruited from a community (Nanjing, Jiangsu, China) from July to September 2018. NAFLD was diagnosed based on *Guideline of prevention and treatment for non-alcoholic fatty liver disease: a 2018 update* (National Workshop on Fatty Liver and Alcoholic Liver Disease, Chinese Society of Hepatology, Association et al., 2018). Diagnostic guidelines: (1) no history of drinking or overdose drinking (less than 210 g/week ethanol for men and 140 g/week for women in the past 12 months); (2) absence of drug hepatitis, hepatitis C virus genotype 3 infection, hepatolenticular degeneration, and other specific diseases that could result in fatty liver; (3) mildly to moderately increased serum levels of transaminase and γ-glutamyl transpeptidase (γ-GT) (<5 times above the upper normal limit), usually presenting as an increase in alanine aminotransferase (ALT); (4) metabolic syndrome constituents, such as visceral obesity, hyperglycemia, blood lipid disorder, and hypertension; (5) imaging results meeting the diagnostic criteria of diffuse fatty liver; and (6) histological findings of liver biopsy meeting the pathological diagnostic criteria of fatty liver disease. Since liver biopsy was difficult to obtain, we used the liver imaging methods mentioned in *the guideline*. NAFLD was diagnosed if criteria 1–4 coexist with criterion 5. The hepatic ultrasound examination was performed using a LOGIQ-E9 ultrasound system (General Electric Healthcare, Milwaukee, WI, United States).

Those patients diagnosed with NAFLD were grouped. The non-NAFLD controls were collected from the same community during the study period and randomly assigned to the control group. The constituent ratios of gender and age between case and control groups were considered similar, according to the result of frequency matching. Included were patients (1) signing informed consent; (2) aged between 18 and 60 years. Excluded were those: (1) taking antihypertensive, antidiabetic, lipid-lowering, or hypouricemic agents within 24-h before physical examination; (2) with infection, acute or chronic gastrointestinal diseases, autoimmune diseases, or malignant tumors; (3) with history of other viral hepatitis, alcoholic liver disease, or primary liver cancer; (4) having excessive alcohol consumption (alcohol consumption ≥ 30 g/day in males and ≥ 20 g/day in females); (5) receiving a liver transplant within the previous year or had complications of advanced liver disease (varicose veins, ascites, etc.); (6) with drug-induced fatty hepatitis; or (7) with a history of psychiatric disorders.

After literature review, we assumed that the frequency of gene mutation in the general population was 10–30%, odds ratio (OR) was 1.5, two-sided test α was 0.05, and power of test (1-β) was 80%. Sample size was estimated by NCSS-PASS 11.0 software (Dawson edition; Kaysville, UT, United States). The sample size of 1,114 NAFLD cases and 1,909 controls in this study was large enough to guarantee the production of reliable results.

The study was performed in accordance with the World Medical Association Declaration of Helsinki on ethical principles in medical research involving human subjects and was approved by the Institutional Ethics Review Committee of Nanjing Medical University (Nanjing, China). Written informed consent was obtained before blood test and genetic analysis.

### Data and Blood Sample Collection

The demographic and clinical characteristics of all participants were collected from self-designed questionnaires and electronic medical records. All participants underwent abdominal ultrasound and blood biochemical tests. Five-milliliter ethylenediaminetetraacetic acid (EDTA) anticoagulant venous blood was collected from the fasting participants *via* the antecubital vein in the morning. The serum and blood cells in each blood sample were separated and frozen at −80°C within 2 h, until further serological tests and genotyping assays.

### SNP Selection and Genotyping

We searched for target SNPs with potential biological function in three candidate genes (*CYP2R1*, *CYP24A1*, and *CYP27B1*). The selection processes were as follows: (1) downloading the genotype databases of *CYP2R1*, *CYP24A1*, and *CYP27B1* in Han Chinese in Beijing (CHB) from the 1,000 Genomes Project database^1^; (2) importing the genotype databases into the Haploview software (version 4.2; Broad Institute, Cambridge, MA, United States). Parameters: Hardy-Weinberg (H-W) *p*-Value cutoff = 0.05; minor allele frequency (MAF) = 0.05; *r*^2^ ≥ 0.8. At this point, 41 tagging SNPs (tagSNPs) were generated; (3) searching for related studies, including (I) relevant research in the Chinese population; (II) studies on NAFLD, hypertension, type 2 diabetes, hyperlipidemia, metabolic syndrome, etc. Definitively, 11 candidate SNPs (*CYP2R1*-rs10741657, rs12794714, rs2060793, and rs1993116; *CYP24A1*-rs2296241, rs2248359, rs927650, and rs6068816; and *CYP27B1*-rs703842, rs10877012, and rs4646536) were selected for further genotyping.

We used magnetic bead method (blood genomic extraction kit; Pangu Genome Nanotechnology Co., Ltd.; Nanjing, China) for isolating genomic DNA from EDTA-anticoagulated blood samples. Genotyping assay was performed with a TaqMan allelic discrimination assay on the Light Cycler 480 II Real-Time PCR System (Roche, Switzerland). Detailed information on primers and probes was shown in Supplementary Table 1. Some measures were implemented to control the data quality as follows: (1) blind methods were adopted in genotyping, so that all technicians were unclear about the clinical data of the participants; and (2) repeated experiments were conducted in 10% of random samples with a repeatability of 100%. Genotyping success rate of all SNPs was above 95%. All tests were carried out in accordance with the manufacturer’s instructions.

### *In silico* Analysis

To further explore the potential functions of gene variants, we performed bioinformatics analysis using some online database as follows: (1) determining the genetic variation-specific location sites on the chromosome and transcriptional regulation information with NCBI dbSNP^2^; (2) viewing the scores of all genetic variation sites in the Regulome DB database,^3^ and SNP scoring 1–3 might act in transcriptional regulation; (3) checking whether the genetic variation sites were located on the histone modification peak through the UCSC Genome Browser database^4^; and (4) using the RNA fold Web Server^5^ to predict the effect of genetic variation of positive SNP on the secondary structure.

### Statistical Analyses

All data analyses were performed using IBM SPSS Statistics for Windows 23.0 (IBM Corp, Armonk, NY, United States) and R software v3.4.3.^6^ Distributions of demographic and clinical characteristics among case and control groups were compared by the Chi-square (χ^2^) test (for categorical variables), Student’s *t*-test, or Mann-Whitney *U*-test (for continuous variables). Logistic regression analysis with adjustment for gender, age, aspartate aminotransferase (AST), ALT, γ-GT, triglyceride (TG), total cholesterol (TC), and high-density lipoprotein cholesterol (HDL-C) was used to calculate odds ratio (OR) and 95% confidence interval (95% CI) for assessing the associations of genotypes with the risk of NAFLD. Each SNP was analyzed using codominant model (mutant homozygous type vs. wild type and heterozygous type vs. wild type, respectively), dominant model (mutant homozygous type + heterozygous type vs. wild type), recessive model (mutant homozygous type vs. heterozygous type + wild type), and additive models (mutant homozygous type vs. heterozygous type vs. wild type). False discovery rate (FDR) corrections were used for multiple comparisons, considering the *p*_FDR_-value ≤ 0.25 as modest confidence that the correlation represented a positive result (Woods et al., 2015). The combined effect of three independent SNPs (*CYP24A1*-rs2296241, *CYP24A1*-rs2248359, and *CYP27B1*-rs4646536) was analyzed using the Cochran-Armitage trend test. Subgroup analysis was performed for positive SNPs, and *Q*-test was performed to calculate the heterogeneity between subgroups. Multivariate stepwise logistic regression analysis was used to determine the independent predictive factors for NAFLD. A receiver-operating characteristic curve (ROC) was used to represent the risk prediction model for NAFLD, with the area under the receiver operating characteristic curve (AUROC) indicating its predictive power. A two-tailed test with a *p*-value < 0.05 was regarded as statistically significant in all analyses.

## Results

### Basic Characteristics of Study Subjects

A total of 3,023 participants were enrolled in our study, including 1,114 NAFLD cases and 1,909 controls. The distribution of demographic and clinical characteristics in two study groups is summarized in Table 1. No significant differences were observed in age and gender between the control and NAFLD groups (all *p* > 0.05). However, there were significant differences in body mass index (BMI), waist circumference (WC), systolic blood pressure (SBP), diastolic blood pressure (DBP), TG, TC, HDL-C, low-density lipoprotein cholesterol (LDL-C), glucose (GLU), γ-GT, ALT, AST, direct bilirubin (DBIL), and total bilirubin (TBIL) (all *p* < 0.001).

**TABLE 1:** Distributions and comparisons of demographic and clinical characteristics between NAFLD case and control groups.

Variables	Controls (*N* = 1,909)	NAFLD cases (*N* = 1,114)	χ^2^/*t*/*Z*	*p*-value
Gender				
Male	1,564 (81.9)	941 (84.5)	3.203	0.073^a^
Female	345 (18.1)	173 (15.5)		
Age (years)				
≤40	1,018 (53.3)	610 (54.8)	0.580	0.446^a^
>40	891 (46.7)	504 (45.2)		
Mean ± SD	39.72 ± 9.63	40.41 ± 8.95	–1.943	0.052^b^
BMI (kg/m^2^)	22.68 ± 2.57	25.41 ± 2.53	–27.867	**<0.001** ^b^
WC (cm)	81.29 ± 8.04	88.76 ± 8.04	–23.933	**<0.001** ^b^
SBP (mmHg)	124.27 ± 13.97	130.53 ± 15.09	–11.335	**<0.001** ^b^
DBP (mmHg)	74.58 ± 9.58	79.23 ± 10.36	–12.263	**<0.001** ^b^
TG (mmol/L)	1.02 (0.78, 1.35)	1.62 (1.17, 2.23)	–23.273	**<0.001** ^c^
TC (mmol/L)	4.42 (3.96, 4.99)	4.71 (4.17, 5.29)	–8.527	**<0.001** ^c^
HDL-C (mmol/L)	1.26 (1.07, 1.47)	1.06 (0.91, 1.22)	–18.307	**<0.001** ^c^
LDL-C (mmol/L)	2.60 (2.21, 3.08)	2.81 (2.28, 3.28)	–5.211	**<0.001** ^c^
GLU (mmol/L)	2.56 (2.30, 4.72)	3.44 (2.36, 4.92)	–6.143	**<0.001** ^c^
γ-GT (U/L)	17 (13, 25)	28 (19, 42)	–20.167	**<0.001** ^c^
ALT (U/L)	17 (13, 24)	27 (19, 40)	–20.367	**<0.001** ^c^
AST (U/L)	18 (16, 22)	21 (17, 26)	–12.429	**<0.001** ^c^
DBIL (μmol/L)	4.30 (3.43, 5.39)	4.02 (3.30, 5.05)	–4.403	**<0.001** ^c^
TBIL (μmol/L)	14.30 (11.34, 18.74)	13.85 (10.82, 17.92)	–2.736	**<0.001** ^c^

*NAFLD, non-alcoholic fatty liver disease; SD, standard deviation; BMI, body mass index; WC, waist circumference; SBP, systolic blood pressure; DBP, diastolic blood pressure; TG, triglyceride; TC, total cholesterol; HDL-C, high-density lipoprotein cholesterol; LDL-C, low-density lipoprotein cholesterol; GLU, glucose; γ-GT, γ-glutamyl transpeptidase; ALT, alanine aminotransferase; AST, aspartate aminotransferase; DBIL, direct bilirubin; TBIL, total bilirubin.*

*Data are presented as mean ± standard deviation, median (interquartile range).*

*Bold type indicates statistically significant results.*

*^*a*^Chi-squared test.*

*^*b*^Student’s t-test.*

*^*c*^Mann-Whitney U-test.*

### Associations Between *CYP2R1*, *CYP24A1*, and *CYP27B1* SNPs and NAFLD Risk

The genotype distributions of the 11 SNPs between the two study groups and the results of the logistic regression analysis are shown in Table 2. After adjusting for gender, age, AST, ALT, γ-GT, TG, TC, and HDL-C, logistic regression analyses showed that *CYP24A1*-rs2296241-A allele (AA vs. GG: adjusted OR = 1.337, 95% CI = 1.035–1.726, *p* = 0.026; recessive model: adjusted OR = 1.316, 95% CI = 1.048–1.653, *p* = 0.018; additive model: adjusted OR = 1.135, 95% CI = 1.002–1.287, *p* = 0.047), *CYP24A1*-rs2248359-T allele (TT vs. CC: adjusted OR = 1.348, 95% CI = 1.037–1.753, *p* = 0.026; recessive model: adjusted OR = 1.315, 95% CI = 1.033–1.674, *p* = 0.026; additive model: adjusted OR = 1.137, 95% CI = 1.002–1.289, *p* = 00.047), and *CYP27B1*-rs4646536-T variant (TT vs. CC: adjusted OR = 1.359, 95% CI = 1.021–1.808, *p* = 0.035; additive model: adjusted OR = 1.147, 95% CI = 1.005–1.310, *p* = 0.042) significantly improved the risk of NAFLD. They were also significant after FDR correcting for multiple comparisons (all *p*_FDR_ ≤ 0.25, Supplementary Table 1). However, no significant association was observed between *CYP2R1*-rs10741657, *CYP2R1*-rs12794714, *CYP2R1*-rs2060793, *CYP2R1*-rs1993116, *CYP24A1*-rs927650, *CYP24A1*-rs6068816, *CYP27B1*-rs703842, and *CYP27B1*-rs10877012 variants (all *p* > 0.05).

**TABLE 2:** Genotype distributions among the two study groups and association analyses of these 11 SNPs and NAFLD.

SNPs	Controls [*n* (%)]	NAFLD cases [*n* (%)]	OR (95% CI)^a^	*p*-value^a^
***CYP2R1*-rs10741657**				
GG	737 (39.7)	421 (38.7)	1.00 (ref.)	
AG	888 (47.8)	530 (48.8)	1.018 (0.843, 1.230)	0.850
AA	233 (12.5)	136 (12.5)	0.996 (0.752, 1.319)	0.978
Dominant model			1.014 (0.847, 1.213)	0.883
Recessive model			0.986 (0.759, 1.281)	0.917
Additive model			1.004 (0.881, 1.143)	0.956
***CYP2R1*-rs12794714**				
GG	706 (37.6)	449 (40.6)	1.00 (ref.)	
AG	933 (49.7)	499 (45.1)	0.865 (0.717, 1.045)	0.133
AA	239 (12.7)	158 (14.3)	1.212 (0.923, 1.593)	0.167
Dominant model			0.916 (0.767, 1.094)	0.330
Recessive model			1.192 (0.928, 1.530)	0.170
Additive model			1.000 (0.880, 1.135)	0.997
***CYP2R1*-rs2060793**				
GG	744 (39.7)	432 (39.7)	1.00 (ref.)	
AG	896 (47.8)	519 (47.7)	0.951 (0.787, 1.149)	0.602
AA	234 (12.5)	138 (12.7)	0.979 (0.741, 1.294)	0.881
Dominant model			0.957 (0.800, 1.145)	0.630
Recessive model			1.006 (0.776, 1.304)	0.964
Additive model			0.979 (0.860, 1.114)	0.744
***CYP2R1*-rs1993116**				
CC	758 (40.4)	439 (40.2)	1.00 (ref.)	
TC	872 (46.5)	511 (46.8)	0.962 (0.797, 1.161)	0.688
TT	247 (13.2)	141 (12.9)	0.950 (0.721, 1.251)	0.716
Dominant model			0.960 (0.803, 1.146)	0.649
Recessive model			0.970 (0.751, 1.253)	0.815
Additive model			0.971 (0.855, 1.104)	0.657
***CYP24A1*-rs2296241**				
GG	655 (35.3)	354 (32.7)	1.00 (ref.)	
AG	904 (48.7)	519 (47.9)	1.026 (0.843, 1.250)	0.796
AA	296 (16.0)	210 (19.4)	**1.337 (1.035, 1.726)**	**0.026**
Dominant model			1.102 (0.915, 1.327)	0.304
Recessive model			**1.316 (1.048, 1.653)**	**0.018**
Additive model			**1.135 (1.002, 1.287)**	**0.047**
***CYP24A1*-rs2248359**				
CC	738 (40.1)	410 (38.5)	1.00 (ref.)	
TC	842 (45.7)	479 (45.0)	1.048 (0.863, 1.272)	0.635
TT	261 (14.2)	175 (16.4)	**1.348 (1.037, 1.753)**	**0.026**
Dominant model			1.119 (0.933, 1.341)	0.225
Recessive model			**1.315 (1.033, 1.674)**	**0.026**
Additive model			**1.137 (1.002, 1.289)**	**0.047**
***CYP24A1*-rs927650**				
CC	1,003 (54.4)	585 (54.1)	1.00 (ref.)	
TC	704 (38.2)	415 (38.4)	1.090 (0.905, 1.312)	0.364
TT	138 (7.5)	82 (7.6)	1.219 (0.870, 1.709)	0.249
Dominant model			1.110 (0.931, 1.324)	0.245
Recessive model			1.176 (0.847, 1.633)	0.332
Additive model			1.098 (0.956, 1.261)	0.187
***CYP24A1*-rs6068816**				
CC	737 (39.5)	435 (39.8)	1.00 (ref.)	
TC	879 (47.1)	509 (46.5)	1.006 (0.833, 1.215)	0.952
TT	250 (13.4)	150 (13.7)	1.145 (0.872, 1.505)	0.329
Dominant model			1.036 (0.867, 1.239)	0.697
Recessive model			1.142 (0.887, 1.470)	0.304
Additive model			1.053 (0.927, 1.197)	0.426
***CYP27B1*-rs703842**				
CC	742 (39.5)	415 (38.0)	1.00 (ref.)	
TC	893 (47.6)	537 (49.1)	1.077 (0.891, 1.302)	0.442
TT	242 (12.9)	141 (12.9)	1.143 (0.863, 1.512)	0.352
Dominant model			1.090 (0.910, 1.306)	0.347
Recessive model			1.096 (0.846, 1.419)	0.490
Additive model			1.071 (0.940, 1.221)	0.303
***CYP27B1*-rs10877012**				
TT	734 (39.0)	413 (37.5)	1.00 (ref.)	
TG	908 (48.2)	546 (49.5)	1.055 (0.874, 1.275)	0.577
GG	242 (12.8)	143 (13.0)	1.115 (0.842, 1.475)	0.448
Dominant model			1.067 (0.891, 1.278)	0.479
Recessive model			1.081 (0.835, 1.400)	0.555
Additive model			1.056 (0.926, 1.203)	0.417
***CYP27B1*-rs4646536**				
CC	735 (39.6)	403 (36.6)	1.00 (ref.)	
TC	908 (48.9)	551 (50.1)	1.102 (0.912, 1.332)	0.315
TT	213 (11.5)	146 (13.3)	**1.359 (1.021, 1.808)**	**0.035**
Dominant model			1.149 (0.958, 1.377)	0.134
Recessive model			1.285 (0.987, 1.673)	0.062
Additive model			**1.147 (1.005, 1.310)**	**0.042**

*CYP2R1, cytochrome P450 family 2 subfamily R member 1 gene; CYP24A1, cytochrome P450 family 24 subfamily A member 1 gene; CYP27B1, cytochrome P450 family 2 subfamily A member 1 gene; SNPs, single nucleotide polymorphisms; NAFLD, non-alcoholic fatty liver disease; OR, odds ratio; CI, confidence interval.*

*A pair of allele such as C/G, if G is a less-frequent allele, then dominant model (CG + GG vs. CC), recessive model (GG vs. CG + CC), and additive model (GG vs. CG vs. CC).*

*Bold type indicates statistically significant results.*

*^*a*^Logistic regression model, adjusted for gender, age, AST, ALT, GGT, TG, TC, and HDL-C.*

### Combined Effects Analysis and Stratified Analysis

The combined effects of rs2296241-A, rs2248359-T, and rs4646536-T on susceptibility to NAFLD were evaluated by counting the number of unfavorable alleles from these three SNPs, as shown in Table 3. The results showed that NAFLD prevalence was increased in people carrying more unfavorable alleles. Compared with those who had “0” unfavorable alleles, participants with “1–3” or “4–6” unfavorable alleles had significantly increased risk of NAFLD (adjusted OR = 1.348, 95% CI = 1.016–1.788, *p* = 0.039; adjusted OR = 1.438, 95% CI = 1.038–1.993, *p* = 0.029, respectively). The more unfavorable alleles, the higher NAFLD risk, suggesting a significant locus-dosage effect of combined alleles on NAFLD risk (*p*_trend_ = 0.049). Moreover, participants carrying more than one unfavorable allele of relevant SNPs had a 36.8% higher risk of NAFLD, compared with people without an unfavorable allele (*p* = 0.028).

**TABLE 3:** Combined effects of rs2296241-A, rs2248359-T, and rs4646536-T on NAFLD risk.

Variables	Controls [*n* (%)]	NAFLD cases [*n* (%)]	NAFLD prevalence (%)	OR (95% CI)	*p*-value^a^
0	244 (12.8)	113 (10.1)	31.7	1.00 (ref.)	
1–3	1,310 (68.6)	773 (69.4)	37.1	**1.348 (1.016, 1.788)**	**0.039**
4–6	355 (18.6)	228 (20.5)	39.1	**1.438 (1.038, 1.993)**	**0.029**
**Trend**					***p*_trend_ = 0.049^b^**
0	244 (12.8)	113 (10.1)	31.7	1.00 (ref.)	
1–6	1,665 (87.2)	1,001 (89.9)	37.5	**1.368 (1.035, 1.807)**	**0.028**

*Variables are numbers of combined unfavorable alleles (rs2296241-A, rs2248359-T, and rs4646536-T).*

*Bold type indicates statistically significant results.*

*^*a*^Logistic regression analyses adjusted for gender, age, AST, ALT, GGT, TG, TC, and HDL-C.*

*^*b*^p_trend_-Value was analyzed by Cochran-Armitage trend test.*

Afterward, stratified analysis was conducted to further evaluate the combined effects of rs2296241-A, rs2248359-T, and rs4646536-T (Supplementary Table 2). We found that the association between unfavorable genotypes and NAFLD risk was still significant in men (adjusted OR = 1.223, 95% CI = 1.029–1.454, *p* = 00.022), γ-GT ≤ 50 U/L (adjusted OR = 1.189, 95% CI = 1.007–1.404, *p* = 0.042), non-low HDL-C (adjusted OR = 1.287, 95% CI = 1.058–1.565, *p* = 0.012). Moreover, heterogeneity test discovered no statistical significance in all the subgroups (all *p* > 0.05).

### Risk Factors of NAFLD

**FIGURE 1:**
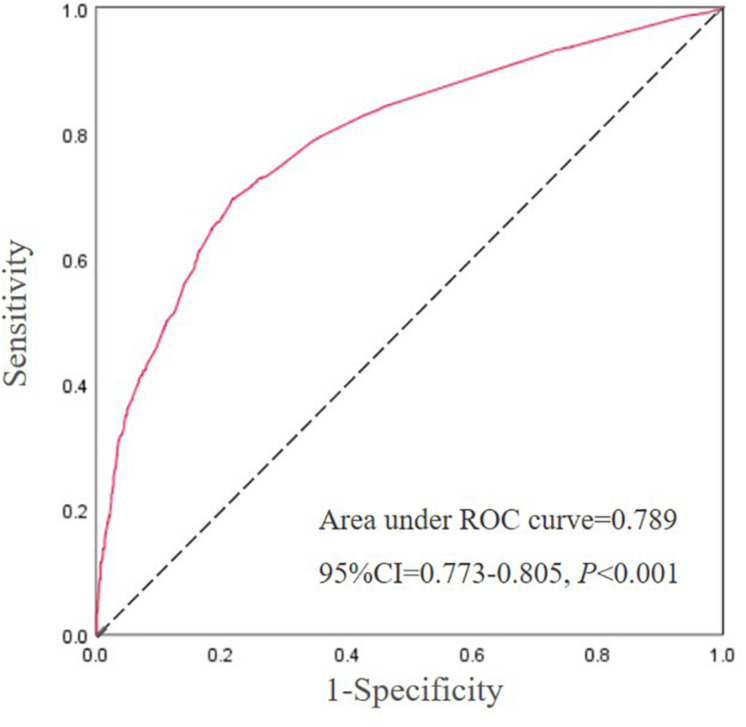
The ROC curve for the influence factors of Table 4. *Abbreviations:* NAFLD, non-alcoholic fatty liver disease; AUROC, area under the receiver operating curve. The response variable is NAFLD risk and the diagnostic test variable is a combination of age, visceral obesity, ALT, γ-GT, hypertriglyceridemia, hypertension, low HDL-C, hyperglycemia, and unfavorable alleles with the coefficients taken from the regression analysis.

A stepwise regression model was established comprising gender, age, visceral obesity, ALT, γ-GT, hypertriglyceridemia, hypertension, low HDL-C, hyperglycemia, and combined unfavorable alleles (*CYP24A1* rs2296241-A, *CYP24A1* rs2248359-T, and *CYP27B1* rs4646536-T). The result showed that age (OR = 0.785, 95% CI = 0.646–0.954, *p* = 0.015), visceral obesity (OR = 4.400, 95% CI = 3.511–5.515, *p* < 0.001), ALT (OR = 2.240, 95% CI = 1.689–2.970, *p* < 0.001), γ-GT (OR = 1.414, 95% CI = 1.031–1.938, *p* = 0.031), hypertriglyceridemia (OR = 3.497, 95% CI = 2.806–4.360, *p* < 0.001), hypertension (OR = 1.723, 95% CI = 1.374–2.160, *p* < 0.001), low HDL-C (OR = 1.722, 95% CI = 1.404–2.111, *p* < 0.001), hyperglycemia (OR = 1.515, 95% CI = 1.016–2.260, *p* = 0.042), and unfavorable alleles (OR = 1.459, 95% CI = 1.093–1.948, *p* = 0.010) were independent influencing predictors of NAFLD in Table 4. Subsequently, we constructed a combined factor model based on the above nine variables for assessing NAFLD risk. The combined model achieved an AUROC of 0.789 (95% CI = 0.773–0.805) (Figure 1). At a cutoff value of −0.85, the sensitivity and specificity of this novel model were 69.6% (66.7–72.4) and 78.1% (76.0–80.0), respectively. The positive likelihood ratio and negative likelihood ratio were 3.18 and 0.39, respectively.

**TABLE 4:** Multivariate stepwise regression analysis on influence factors for NAFLD risk.

Variables	β	SE	Wald	OR (95% CI)	*p*-value
Gender (male vs. female)	0.246	0.126	3.774	1.278 (0.998, 1.638)	0.052
Age (≤ 40 vs. > 40 years)	–0.242	0.100	5.919	**0.785 (0.646, 0.954)**	**0.015**
Visceral obesity	1.482	0.115	165.332	**4.400 (3.511, 5.515)**	**< 0.001**
ALT (≤ 40 vs. > 40 U/L)	0.806	0.144	31.356	**2.240 (1.689, 2.970)**	**< 0.001**
γ-GT (≤ 50 vs. > 50 U/L)	0.346	0.161	4.632	**1.414 (1.031, 1.938)**	**0.031**
Hypertriglyceridemia (no vs. yes)	1.252	0.112	124.003	**3.497 (2.806, 4.360)**	**< 0.001**
Hypertension (no vs. yes)	0.544	0.115	22.176	**1.723 (1.374, 2.160)**	**< 0.001**
Low HDL-C (no vs. yes)	0.543	0.104	27.250	**1.722 (1.404, 2.111)**	**< 0.001**
Hyperglycemia (no vs. yes)	0.416	0.204	4.155	**1.515 (1.016, 2.260)**	**0.042**
Unfavorable alleles	0.378	0.147	6.578	**1.459 (1.093, 1.948)**	**0.010**
Cons	–7.629	0.500	232.616		

*ALT, alanine aminotransferase; γ-GT, γ-glutamyl transpeptidase; HDL-C, high-density lipoprotein cholesterol; Cons, constant term; OR, odds ratio; CI, confidence interval.*

*Bold type indicates statistically significant results.*

### Functions of Positive SNPs

**FIGURE 2:**
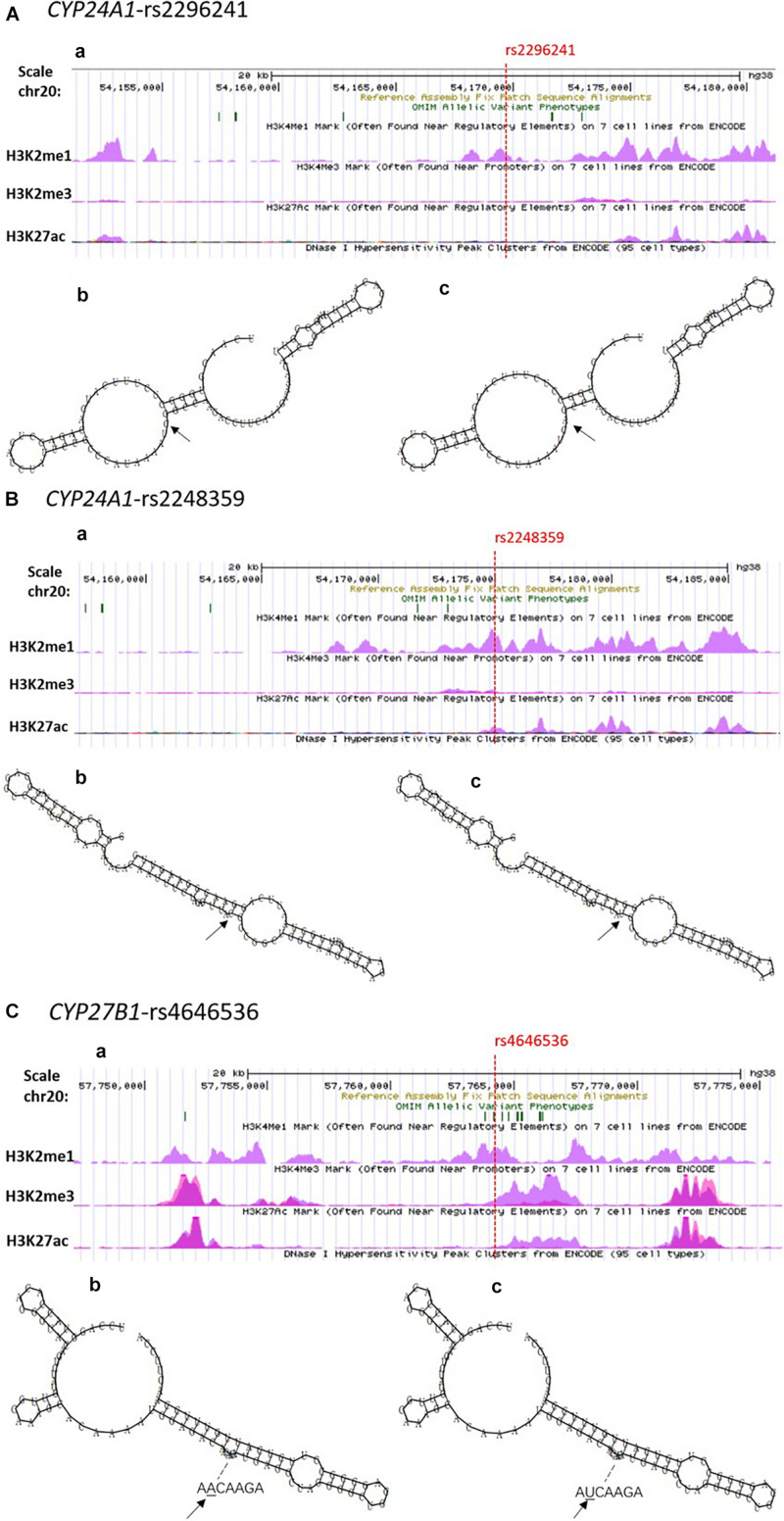
Functional prediction of positive SNPs in *CYP24A1* and *CYP27B1*. Notes. **(A)** *CYP24A1*-rs2296241; **(B)** *CYP24A1*-rs2248359; **(C)** *CYP27B1*-rs4646536. **(a)** USCS functional annotations of SNPs, respectively. The red dotted line indicated the position of target SNPs (available at http://genome.ucsc.edu/). **(b**,**c)** Influence of SNPs on the mRNA secondary structures. **(b)** Wild-type sequence; **(c)** mutant-type sequence. The arrows indicate the position of the mutation (50 bases upstream and 50 bases downstream of the mutation). The minimum free energies (MFE) for the G and A allele of rs2296241 were estimated at −10.9 and −7.5 kcal/mol, respectively. The MFE for the C and T allele of rs2248359 were estimated at −21.5 and −18.6 kcal/mol, respectively. The MFE for the A and T alleles of rs4646536 were all estimated at −30.5 kcal/mol by RNA fold Wed Server.

The Regulome DB score for the *CYP24A1*-rs2296241, *CYP24A1*-rs2248359, and *CYP27B1*-rs4646536 were 4, 4, and 1d, respectively.^7^ The UCSC prediction showed that rs2296241, rs2248359, and rs4646536 were all enriched near the H3K4Me1 marker (Figure 2). The effect of rs2296241, rs2248359, and rs4646536 predicted by RNA fold Web Server on the secondary structure of *CYP24A1* and *CYP27B1* mRNA is shown in Figure 2. The arrows indicated the position of the mutation (50 bases upstream and 50 bases downstream of the mutation). The minimum free energy (MFE) of G and A alleles of *CYP24A1*-rs2296241 were estimated at −10.9 and −7.5 kcal/mol, respectively. The MFE of C and T alleles of *CYP24A1*-rs2248359 were estimated at −21.5 and −18.6 kcal/mol, respectively. The MFE of A and T alleles of *CYP27B1*-rs4646536 were all estimated at −30.5 kcal/mol.

## Discussion

In this case-control study, we explored the associations of vitamin D family genes *CYP2R1*, *CYP24A1*, and *CYP27B1* genetic polymorphism with the risk of NAFLD among Chinese Hans. Our result showed that *CYP24A1* rs2296241-A, rs2248359-T, and *CYP27B1* rs4646536-T as unfavorable alleles were associated with the increased risk of NAFLD. The combination of clinical factors and unfavorable alleles exhibited a desirable predictive value for the risk of NAFLD.

NAFLD is a complex metabolic disorder closely associated with obesity, T2DM, and metabolic syndrome (Younossi, 2019). Vitamin D is a pleiotropic hormone definitively involved in immune-inflammatory and metabolic processes (Charoenngam and Holick, 2020). Accumulative previous studies have suggested vitamin D deficiency highly prevalent among the general Asian population (Cheng et al., 2017; Cho et al., 2019). Several recent meta-analyses have suggested that low vitamin D level may impact disease progression of NAFLD, while in chronic liver diseases, enzymatic conversion (hydroxylation) is disrupted in the liver through a variety of mechanisms, leading to low serum vitamin D levels (Zhu and DeLuca, 2012; Zhang et al., 2019; Liu et al., 2020). SNPs in genes involved in the vitamin D metabolic process could affect vitamin D status. The associations of *CYP2R1*, *CYP24A1*, and *CYP27B1* gene polymorphisms with vitamin D deficiency in multiple population have been reported (Gibson et al., 2018; Arai et al., 2019). In this study, we genotyped three target genes (*CYP2R1*, *CYP24A1*, and *CYP27B1*) encoding hydroxylase in the vitamin D metabolic pathway. No relationship was found between *CYP2R1* and NAFLD. Similar negative results were found in previous studies among different populations (Gibson et al., 2018). However, positive results have shown that genetic variants of *CYP24A1* and *CYP27B1* can increase the risk of NAFLD. To our knowledge, this is the first study revealing a relationship between *CYP24A1* and *CYP27B1* SNPs and NAFLD risk.

Although no studies have shown a relationship between the polymorphisms of *CYP24A1*, *CYP27B1*, and NAFLD, these variants have been extensively investigated in other diseases, such as organ-specific autoimmune endocrine diseases (Ma et al., 2020), CVD (Qian et al., 2020), metabolic diseases (Yu et al., 2020), and multiple cancers (Hibler et al., 2015; Torkko et al., 2020). Our results also confirmed that NAFLD patients with a combined load of unfavorable alleles (rs2296241-A, rs2248359-T, and rs4646536-T) exhibited an association with the increase in NAFLD risk, and this association was also in a dose-dependent manner. These findings would provide useful information of risk assessment or possible diagnostic markers for NAFLD.

In the model of combined unfavorable alleles with other clinical factors (binary age, visceral obesity, ALT, γ-GT, hypertriglyceridemia, hypertension, low HDL-C, hyperglycemia), gender was not significant, but the remaining nine were independent risk factors of NAFLD. These results are almost consistent with NAFLD guidelines and some previous studies. Abnormal ALT and γ-GT levels, hypertriglyceridemia, hypertension, low HDL-C levels, and hyperglycemia have been recognized as predictors of metabolic syndrome (National Workshop on Fatty Liver and Alcoholic Liver Disease, Chinese Society of Hepatology, Association et al., 2018). Age and BMI are established predictors of liver fibrosis and have been included in the NAFLD fibrosis score formula (Angulo et al., 2007). WC and γ-GT are included in the non-invasive model Fatty Liver Index (FLI) for risk prediction of NAFLD (Bedogni et al., 2006). Moreover, the AUROC of our model combining the above nine variables was 0.789, indicating that it had a desirable predictive value. Given its potential predictive value, the predictive model combining clinical and genetic factors might provide new avenue for early screening in high-risk population. Cost-benefit analysis should be considered in future studies.

In this study, we used multiple bioinformatics databases to predict the function of positive SNPs. The Regulome DB score for the *CYP24A1*-rs2296241, *CYP24A1*-rs2248359, and *CYP27B1*-rs4646536 were 4, 4, and 1 days, respectively, which indicated that these loci have strong potential functions, regulate the expression of *CYP24A1* and *CYP27B1* by changing multiple regulatory motifs, and interfere with protein-binding activity (Boyle et al., 2012). The performances of UCSC showed that rs2296241, rs2248359, and rs4646536 were involved in promoter and enhancer modification in different cell lines, especially in the vicinity of enhancer elements (H3K2me1 marker) of multiple cell lines. Moreover, they were also related to the change of transcription factor-binding module. In addition, the MFEs of rs2296241 G and A alleles (−10.9 vs. −7.5 kcal/mol) and rs2248359 C and T alleles (−21.5 vs. −18.6 kcal/mol) were different, suggesting that mutation of rs2296241 and rs2248359 may affect the transcription of *CYP24A1*. Further research is warranted to identify the function of these polymorphisms in vitamin D metabolic pathway.

Several potential limitations need to be considered. Firstly, this is a single-center study, and population selection is under-represented. In response, gender and age were matched in the design stage, and multivariate analysis and stratified analysis were carried out to control the influence of the confounding factors. Secondly, we only chose three hydroxylase genes in the vitamin D metabolic pathway, which may not fully analyze the relationship between genetic variants and NAFLD risk. More genetic loci are needed to confirm the effect of genetic variation on the risk of NAFLD. It is necessary to further explore the impact of polygenic loci and their combination with other environmental factors on NAFLD risk in a multicenter population of different races.

## Conclusion

*CYP24A1* (rs2296241, rs2248359) and *CYP27B1* (rs4646536) variants are associated with a high risk of NAFLD in the Chinese Han population. The combination of unfavorable SNPs and metabolic-related indicators shows high efficiency in predicting the risk of NAFLD. These findings might provide new insight into NAFLD pathogenesis and a new tool for early screening of high-risk population.

## Data Availability Statement

The original contributions presented in the study are included in the article/Supplementary Material, further inquiries can be directed to the corresponding author/s.

## Ethics Statement

The studies involving human participants were reviewed and approved by the Institutional Ethics Review Committee of Nanjing Medical University (Nanjing, China). The patients/participants provided their written informed consent to participate in this study.

## Author Contributions

JW designed and organized the study. MXW, RZ, MW, LZ, YD, ZT, HLW, WZ, and YC contributed to the planning, designing, and analyses of the data collection and quality control. MW, RZ, MW, LZ, YD, and ZT performed data reduction and statistical analysis. HLW and WZ provided materials and analysis tools. MXW, RZ, and JW wrote and critically revised the manuscript. All authors read and approved the final manuscript.

## Conflict of Interest

The authors declare that the research was conducted in the absence of any commercial or financial relationships that could be construed as a potential conflict of interest.

## Publisher’s Note

All claims expressed in this article are solely those of the authors and do not necessarily represent those of their affiliated organizations, or those of the publisher, the editors and the reviewers. Any product that may be evaluated in this article, or claim that may be made by its manufacturer, is not guaranteed or endorsed by the publisher.

## Footnotes

1
http://www.1000genomes.org/

2
https://www.ncbi.nlm.nih.gov/snp/

3
https://www.regulomedb.org/

4
https://genome.ucsc.edu/

5
http://rna.tbi.univie.ac.at//cgi-bin/RNAWebSuite/RNAfold.cgi

6
http://www.r-project.org/

7
http://www.regulomedb.org/results
