# Corrigendum: Genetic Polymorphism of Vitamin D Family Genes *CYP2R1, CYP24A1*, and *CYP27B1* Are Associated With a High Risk of Non-alcoholic Fatty Liver Disease: A Case-Control Study

**DOI:** 10.3389/fgene.2021.762978

**Published:** 2021-09-16

**Authors:** Minxian Wang, Ru Zhang, Min Wang, Liuxin Zhang, Yajie Ding, Zongzhe Tang, Hongliang Wang, Wei Zhang, Yue Chen, Jie Wang

**Affiliations:** ^1^Department of Fundamental and Community Nursing, School of Nursing, Nanjing Medical University, Nanjing, China; ^2^The Affiliated Brain Hospital of Nanjing Medical University, Nanjing, China; ^3^Ninghai Road Community Health Service Center of Nanjing, Nanjing, China; ^4^Department of Epidemiology, Shanghai Cancer Institute, Shanghai, China

**Keywords:** non-alcoholic fatty liver disease, cytochrome P450 family 24 subfamily A member 1 gene, cytochrome P450 family 27 subfamily B member 1 gene, polymorphism, risk

In the published article, there was an error regarding the affiliations for Hongliang Wang and Wei Zhang. For author Hongliang Wang, instead of Affiliation 2 “The Affiliated Brain Hospital of Nanjing Medical University, Nanjing, China,” it should be affiliation 3 “Ninghai Road Community Health Service Center of Nanjing, Nanjing, China**”** and for author Wei Zhang, instead of affiliation 3 **“**Ninghai Road Community Health Service Center of Nanjing, Nanjing, China,” it should be affiliation 4 “Department of Epidemiology, Shanghai Cancer Institute, Shanghai, China.”

The authors apologize for this error and state that this does not change the scientific conclusions of the article in any way. The original article has been updated.

## Publisher's Note

All claims expressed in this article are solely those of the authors and do not necessarily represent those of their affiliated organizations, or those of the publisher, the editors and the reviewers. Any product that may be evaluated in this article, or claim that may be made by its manufacturer, is not guaranteed or endorsed by the publisher.

