# Supplementary material for: Genetic Polymorphism of Vitamin D Family Genes CYP2R1, CYP24A1, and CYP27B1 Are Associated With a High Risk of Non-alcoholic Fatty Liver Disease: A Case-Control Study
Source: Front Genet. 2021 Aug 16;12:717533. doi: 10.3389/fgene.2021.717533 (PMC8415785; doi:10.3389/fgene.2021.717533)
Supplement: Supplementary file 1 [file Data_Sheet_1.docx]

**Supplementary Table 1.** Associations of 11 SNPs with NAFLD risk in dominant, recessive, and additive models of multivariable analyses

| SNPs | Co-dominant model  (CC vs. CG) | | Co-dominant model  (CC vs. GG) | | Dominant model  (CG+GG vs. CC) | | Recessive model  (GG vs. CG+CC) | | Additive model  (GG vs CG vs CC) | |
| --- | --- | --- | --- | --- | --- | --- | --- | --- | --- | --- |
|  | *P* | *P*_FDR_^*^ | *P* | *P*_FDR_^*^ | *P* | *P*_FDR_^*^ | *P* | *P*_FDR_^*^ | *P* | *P*_FDR_^*^ |
| rs10741657 (G>A) | 0.850 | 0.935 | 0.978 | 0.978 | 0.883 | 0.883 | 0.917 | 0.964 | 0.956 | 0.956 |
| rs12794714 (G>A) | 0.134 | 0.935 | 0.167 | 0.459 | 0.453 | 0.753 | 0.037 | 0.136 | 0.617 | 0.803 |
| rs2060793 (G>A) | 0.602 | 0.935 | 0.881 | 0.969 | 0.630 | 0.767 | 0.964 | 0.964 | 0.744 | 0.818 |
| rs1993116 (C>T) | 0.688 | 0.935 | 0.716 | 0.875 | 0.649 | 0.767 | 0.815 | 0.964 | 0.657 | 0.803 |
| rs2296241 (G>A) | 0.796 | 0.935 | 0.026 | **0.128** | 0.304 | 0.753 | 0.018 | **0.136** | 0.047 | **0.172** |
| rs2248359 (C>T) | 0.635 | 0.935 | 0.026 | **0.128** | 0.225 | 0.753 | 0.026 | **0.136** | 0.047 | **0.172** |
| rs927650 (C>T) | 0.364 | 0.935 | 0.249 | 0.548 | 0.245 | 0.753 | 0.332 | 0.609 | 0.187 | 0.514 |
| rs6068816 (C>T) | 0.952 | 0.952 | 0.329 | 0.553 | 0.697 | 0.767 | 0.304 | 0.609 | 0.426 | 0.669 |
| rs703842 (C>T) | 0.442 | 0.935 | 0.352 | 0.553 | 0.347 | 0.753 | 0.490 | 0.763 | 0.303 | 0.667 |
| rs10877012 (T>G) | 0.577 | 0.935 | 0.448 | 0.616 | 0.479 | 0.753 | 0.555 | 0.763 | 0.417 | 0.669 |
| rs4646536 (C>T) | 0.315 | 0.935 | 0.035 | **0.128** | 0.134 | 0.753 | 0.062 | **0.171** | 0.042 | **0.172** |

**Notes.**

*Abbreviations*: SNPs, single nucleotide polymorphisms; FDR, false discovery rate.

Bold type indicates statistically significant results, deriving from logistic regression analyses with adjustment for gender, age, AST, ALT, GGT, TG, TC, HDL-C.

^*^ Adjusted *P*-value using FDR for multiple testing correction. The *P*_FDR_ value ≤ 0.25 was regarded as modest confidence that the correlation represented a positive result ^[29]^.

**Supplementary Table 2** Stratified analysis of the association between combined unfavorable alleles and NAFLD risk

| Subgroup | Controls  n (0/1-3/4-6) | NAFLD cases  n (0/1-3/4-6) | OR (95%CI) ^a^ | *P*^a^ | *P*^b^ |
| --- | --- | --- | --- | --- | --- |
| Gender |  |  |  |  |  |
| Male | 197/1085/282 | 94/654/193 | **1.223(1.029, 1.454)** | **0.022** | 0.239 |
| Female | 47/225/73 | 19/119/35 | 0.962(0.655, 1.412) | 0.842 |  |
| Age(years) |  |  |  |  |  |
| ≤40 | 127/714/177 | 59/431/120 | 1.218(0.977, 1.518) | 0.079 | 0.607 |
| >40 | 117/596/178 | 54/342/108 | 1.121(0.896, 1.401) | 0.317 |  |
| Visceral obesity |  |  |  |  |  |
| No | 198/1081/308 | 59/436/125 | 1.149(0.949, 1.391) | 0.155 | 0.467 |
| Yes | 26/134/28 | 47/303/91 | 1.349(0.944, 1.928) | 0.101 |  |
| ALT (U/L) |  |  |  |  |  |
| ≤40 | 214/1123/315 | 75/593/169 | 1.152(0.975, 1.361) | 0.097 | 0.803 |
| >40 | 18/88/20 | 38/178/59 | 1.222(0.812, 1.839) | 0.337 |  |
| AST(U/L) |  |  |  |  |  |
| ≤40 | 228/1191/329 | 106/742/214 | 1.165(0.994, 1.365) | 0.059 | 0.501 |
| >40 | 4/20/6 | 7/29/14 | 2.309(0.723, 7.374) | 0.158 |  |
| γ-GT(U/L) |  |  |  |  |  |
| ≤50 | 219/1128/316 | 89/6333/183 | **1.189(1.007, 1.404)** | **0.042** | 0.677 |
| >50 | 11/79/19 | 24/138/45 | 1.067(0.657, 1.733) | 0.792 |  |
| Hypertriglyceridemia |  |  |  |  |  |
| No | 213/1150/306 | 56/417/117 | 1.173(0.971, 1.418) | 0.098 | 1.000 |
| Yes | 31/160/49 | 57/356/111 | 1.173(0.878, 1.567) | 0.281 |  |
| Hypertension |  |  |  |  |  |
| No | 196/1125/312 | 82/554/163 | 1.158(0.968, 1.385) | 0.109 | 0.508 |
| Yes | 48/185/43 | 31/219/65 | 1.327(0.948, 1.858) | 0.099 |  |
| Low HDL-C |  |  |  |  |  |
| No | 196/1046/269 | 54/409/120 | **1.287(1.058, 1.565)** | **0.012** | 0.204 |
| Yes | 48/264/86 | 59/364/108 | 1.046(0.809, 1.353) | 0.732 |  |
| Hyperglycemia |  |  |  |  |  |
| No | 235/1269/339 | 105/703/210 | 1.156(0.983, 1.361) | 0.080 | 0.905 |
| Yes | 9/41/16 | 8/70/18 | 1.207(0.642, 2.269) | 0.559 |  |

**Notes.**

Combined unfavorable alleles: *CYP24A1* rs2296241-A, *CYP24A1* rs2248359-T and *CYP27B1* rs4646536-T

^a^ Logistic regression model, adjusted for gender, age, AST, ALT, GGT, TG, TC, HDL. (Exclusion of stratification factors)

^b^ *P*-value for the heterogeneity test
